# The Neuropeptide Allatostatin A Regulates Metabolism and Feeding Decisions in *Drosophila*

**DOI:** 10.1038/srep11680

**Published:** 2015-06-30

**Authors:** Julie L. Hentze, Mikael A. Carlsson, Shu Kondo, Dick R. Nässel, Kim F. Rewitz

**Affiliations:** 1 Department of Science, Systems and Models, Roskilde University, Universitetsvej 1, Roskilde 4000, Denmark; 2 Department of Biology, University of Copenhagen, Universitetsparken 15, Copenhagen 2100, Denmark; 3 Department of Zoology, Stockholm University, Svante Arrhenius väg 18B, Stockholm 106 91, Sweden; 4 Genetic Strains Research Center, National Institute of Genetics, Mishima, Shizuoka, Japan

## Abstract

Coordinating metabolism and feeding is important to avoid obesity and metabolic diseases, yet the underlying mechanisms, balancing nutrient intake and metabolic expenditure, are poorly understood. Several mechanisms controlling these processes are conserved in *Drosophila*, where homeostasis and energy mobilization are regulated by the glucagon-related adipokinetic hormone (AKH) and the *Drosophila* insulin-like peptides (DILPs). Here, we provide evidence that the *Drosophila* neuropeptide Allatostatin A (AstA) regulates AKH and DILP signaling. The AstA receptor gene, *Dar-2*, is expressed in both the insulin and AKH producing cells. Silencing of *Dar-2* in these cells results in changes in gene expression and physiology associated with reduced DILP and AKH signaling and animals lacking *AstA* accumulate high lipid levels. This suggests that AstA is regulating the balance between DILP and AKH, believed to be important for the maintenance of nutrient homeostasis in response to changing ratios of dietary sugar and protein. Furthermore, *AstA* and *Dar-2* are regulated differentially by dietary carbohydrates and protein and AstA-neuronal activity modulates feeding choices between these types of nutrients. Our results suggest that AstA is involved in assigning value to these nutrients to coordinate metabolic and feeding decisions, responses that are important to balance food intake according to metabolic needs.

Imbalance between the amount and type of nutrients consumed and metabolized can cause obesity. It is therefore important to understand how animals maintain energy balancing, which is determined by mechanisms that guide feeding decisions according to the internal nutritional status. The fruit fly *Drosophila melanogaster* has become an important model for studies of feeding and metabolism, as the regulation of metabolic homeostasis is conserved from flies to mammals^1^,^2^. In *Drosophila*, hormones similar to insulin and glucagon regulate metabolic programs and nutrient homeostasis. Adipokinetic hormone (AKH) is an important metabolic hormone and considered functionally related to human glucagon and a key regulator of sugar homeostasis^3^. The release of AKH promotes mobilization of stored energy from the fat body, the equivalent of the mammalian liver and adipose tissues^3^,^4^,^5^. Neuroendocrine cells in the corpus cardiacum (CC) express and release AKH^3^ that binds to the AKH receptor (AKHR), a G-protein coupled receptor (GPCR) expressed mainly in the fat body, and promotes mobilization of stored sugar and fat^6^,^7^. Insulin and glucagon have opposing effects important to maintain balanced blood glucose levels. The *Drosophila* genome contains 7 genes coding for insulin-like peptides (DILPs), called *dilp1-7*^8^, which are homologous to the mammalian insulin and insulin-like growth factors (IGFs). The seven DILPs are believed to act through one ortholog of the human insulin receptor that activates conserved intracellular signaling pathways^8^,^9^,^10^. The DILPs are important regulators of metabolism, sugar homeostasis^11^,^12^ and cell growth^13^,^14^. DILP2, 3 and 5 are produced in 14 neurosecretory cells in the brain; the insulin producing cells (IPCs). Genetic ablation of the IPCs results in a diabetic phenotype, increased lifespan and reduced growth^12^,^13^. Because of the growth promoting effects, the activity of the DILPs is tightly linked to dietary amino acid concentrations.

Although metabolism has been extensively studied, the mechanisms that coordinate metabolism and feeding decisions to maintain energy balancing are poorly understood. Neuropeptides are major regulators of behavior and metabolism in mammals and insects^15^,^16^ making them obvious candidates to coordinate these processes. Peptides with a FGL-amide carboxy terminus, called type A allatostatins, have previously been related to feeding and foraging behavior^17^,^18^. Four *Drosophila* Allatostatin A (AstA) peptides have been identified^19^ that are ligands for two GPCRs, the *Drosophila* Allatostatin A receptors DAR-1 and DAR-2^20^,^21^. AstA peptides were originally identified as inhibitors of juvenile hormone (JH) synthesis from the corpora allata (CA) of the cockroach *Diploptera punctata*^22^. However, recently it was shown that AstA does not regulate JH in *Drosophila*^23^. Moreover, DAR-1 and DAR-2 are homologs of the mammalian galanin receptors^24^,^25^, known to be involved in both feeding behavior and metabolic regulation^26^.

We examined the function of AstA in *Drosophila* in an effort to determine whether it is involved in the neuroendocrine mechanisms coupling feeding behavior to metabolic pathways that manage energy supplies. Our data suggest that AstA is a modulator of AKH and DILP signaling. *Dar-2* is expressed in both the IPCs and the AKH producing cells (APCs) of the CC. Silencing of *Dar-2* in the APCs or IPCs resulted in changes in expression of genes associated with reduced AKH or DILP signaling, respectively. Moreover, loss of *AstA* is associated with increased fat body lipid levels, resembling the phenotype of mutants in the DILP and AKH pathways. We also investigated the connection between nutrients and AstA signaling, and found that *AstA* and *Dar-2* are regulated differently in response to dietary carbohydrates and protein, and that activation of AstA-neurons increases the preference for a protein rich diet, while *AstA* loss enhances sugar consumption. Our results suggest that AstA is a key coordinator of metabolism and feeding behavior.

## Results

### *Dar-2* is expressed in the APCs and IPCs

**Figure 1:**
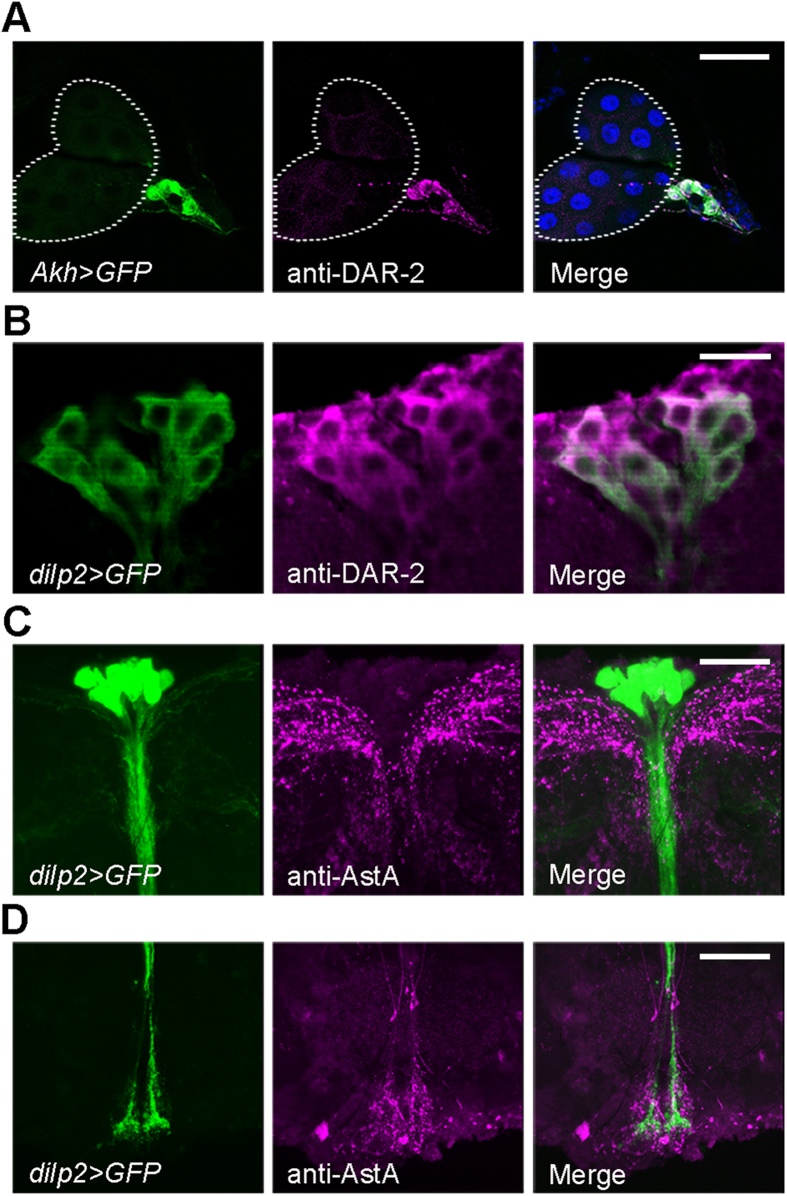
*Dar-2* is expressed in APCs and IPCs and AstA neurites terminate close to the IPCs. (**A**) APCs from 3^rd^ instar larvae expressing *GFP* under control of *Akh>* (green), were stained with an anti-DAR-2 antibody (magenta) and shows co-localization of DAR-2 and GFP. Dotted white lines encircle the prothoracic gland and nuclei are stained by DAPI (blue). (**B**) Immunostaining detects DAR-2 (magenta) in the IPCs of adults labeled by *Dilp2>* driven GFP (green). (**C** and **D**) Staining of adult brains with an AstA antibody (magenta) shows AstA-positive processes terminating in the proximity of the IPCs, visualized by *Dilp2>* driven GFP (green) in the protocerebrum (**C**) and SOG (**D**) of adults. The neuronal processes shown in D are derived from the same cells as shown in C. Scale bars, 50 μm in A, 20 μm in B and 40 μm in C,D.

To investigate the functional role of AstA, we examined the expression of *AstA* and its receptor *Dar-2* in *Drosophila.* Immunostaining of 3^rd^ instar larvae, using a DAR-2 antibody, revealed that *Dar-2* is expressed specifically in a small population of cells at the base of the ring gland, corresponding to the location of the CC, like previously reported^27^. To confirm that the expression was specific for the APCs, we used the APC-specific *Akh-Gal4* (*Akh*>) driver to express *UAS-GFP* (*GFP*) and show that anti-DAR-2 labeling co-localizes with the *GFP* expression in the APCs of the 3^rd^ instar larvae (Fig. 1A). To further support this, we expressed *GFP* using transgenic animals carrying *Gal4* under control of a 4 kb promoter-element comprising the region upstream *Dar-2* (*Dar-2*>) between the transcriptional start codon and the next gene *CG10000*. We observed overlap between GFP and anti-AKH antibody staining in *Dar-2* *>* *GFP* animals (Fig. S1a). Moreover, we also found *Dar-2* expression in the APCs of adults using both DAR-2 antibody staining and *Dar-2* > *GFP*, even though GFP was only observed in a subset of AKH-positive cells. This could be due to the nature of the *Dar-2* promoter construct or that the CC is a heterogeneous cell population and not all cells express the receptor at high levels (Fig. S1b,c). To confirm expression of *Dar-2* in the adult CC, we measured *Dar-2* transcripts in the adult CC and found that the level of *Dar-2* transcript was efficiently reduced using the CC-specific *Akh>* driver in combination with *Dar-2-RNAi* (Fig S1d).

We also observed anti-DAR-2 staining in a population of neurons in the brain anatomically resembling the IPCs (Fig. S2a). To demonstrate that these DAR-2 positive neurons correspond to the IPCs, we used *dilp2-Gal4 (dilp2>)* to drive IPC-specific *GFP* expression and found co-localization with the anti-DAR-2 immunolabeling (Fig. 1B). We also confirmed that *Dar-2>* drives *GFP* expression in the IPCs using a DILP2 antibody that specifically labels the IPCs (Fig. S2b,c). To confirm expression of *Dar-2* in the IPCs, we used the newly developed CRISPR/Cas9 technique to create a T2A-Gal4 reporter knock-in C-terminally in *Dar-2* to tag the endogenous gene. This drives expression of *Gal4* in the same pattern as the endogenous *Dar-2* gene and by insertion of an intervening T2A sequence between *Dar-2* and *Gal4*, the Gal4 can be translated independently of *Dar-2*, which allows Gal4 to enter the nucleus and activate transcription of *UAS-GFP*. This line with a reporter on the endogenous *Dar-2* gene drives expression in DILP2 positive cells in the brain (Fig. S2e), demonstrating *Dar-2* expression in the IPCs. Together these results show that *Dar-2* is expressed in the IPCs of both the larvae and adults.

While the CC cells could be targeted by AstA released from endocrine cells of the gut, the IPCs are located in the brain suggesting that these neurons are regulated by a brain source of AstA. To examine this possibility, we used an AstA antibody to investigate if the neurites of the AstA producing neurons may contact the IPCs. In combination with the visualization of the IPCs (*dilp2* > *GFP*), we found that varicose processes from the AstA neurons terminate in the vicinity of the presumed dendrites of the IPCs in the protocerebrum (Fig. 1C, S2d, S3a) and the IPC processes that branch in the tritocerebrum and subesophageal ganglion (SOG) (Fig. 1D, S3b) of both larvae and adults. Proximity between the AstA-immunoreactive varicosities and the IPCs, expressing *Dar-2*, was also seen with double labeling of anti-AstA and *Dar-2* > *GFP* (Fig. S3c,d). The proximity of the AstA-positive varicosities and the IPCs together with the expression of *Dar-2* in the IPCs and the APCs indicate that AstA may be involved in regulation of metabolism through modulation of DILP and AKH signaling.

### AstA regulates neuroendocrine signaling involved in energy metabolism

**Figure 2:**
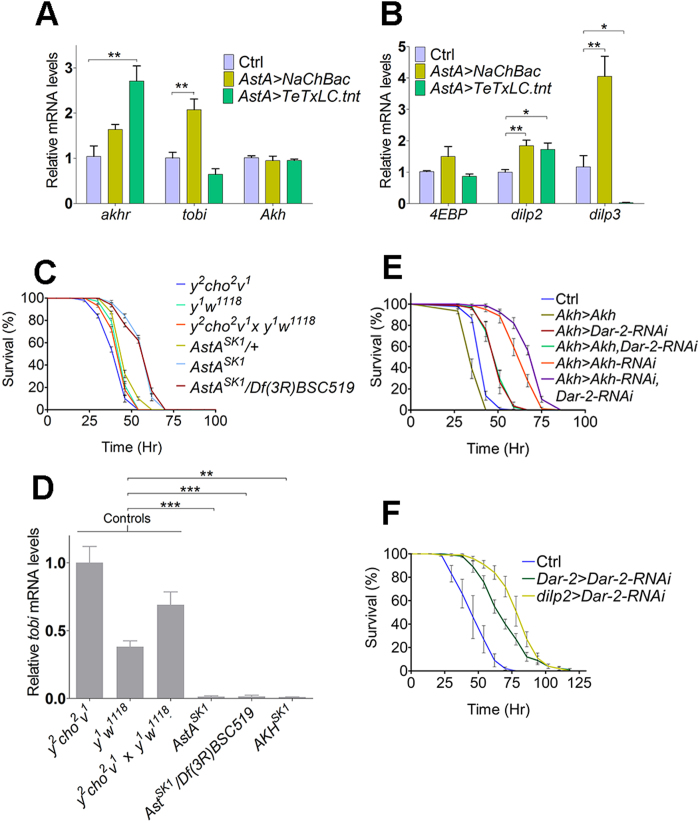
AstA regulates expression of genes related to AKH and DILP signaling and starvation resistance. (**A**,**B**) Expression of key metabolic genes related to AKH (**A**) and DILP (**B**) signaling in flies with increased (*AstA* > *NaChBac*) or decreased (*AstA* > *TeTxLC.tnt*) AstA neuron activity. Changes in expression of several DILP and AKH related genes in *AstA* > *NaChBac* and *AstA* > *TeTxLC.tnt* animals, indicate that AstA affects DILP and AKH signaling. Ctrl: *AstA-Gal4/+*. (**C**) Starvation resistance in flies lacking *AstA* (*AstA*^*SK1*^ and *AstA*^*SK1*^*/Df(3R)BSC519*) compared to *AstA*^*SK1*^/+ heterozygous flies and controls (*y*^*2*^ *cho*^*2*^ *v*^*1*^, *y*^*1*^ *w*^*1118*^ and *y*^*2*^ *cho*^*2*^ *v*^*1*^ x *y*^*1*^ *w*^*1118*^: offspring from crossing these lines). *AstA*^*SK1*^ and *AstA*^*SK1*^*/Df(3R)BSC519* flies were significantly starvation resistant compared to *AstA*^*SK1*^/+ heterozygous flies and controls (logrank test: *P* < 0.0001). (**D**) Expression of the AKH and DILP target gene *tobi* in flies lacking *AstA* (*AstA*^*SK1*^ and *AstA*^*SK1*^*/Df(3R)BSC519*) or *Akh* (*Akh*^*SK1*^). Controls are the same as in C. Error bars indicate standard errors (n = 3–5) (**E**) Starvation resistance in response to *Akh* overexpression (*Akh* > *Akh*), *Dar-2* knock down (*Akh* > *Dar-2-RNAi*), *Dar-2* knock down and combined with overexpression of *Akh* (*Akh* > *Akh*, *Dar-2-RNAi*), *Akh* knock down (*Akh* > *Akh-RNAi*) or *Akh* and *Dar-2* knocked down simultaneously (*Akh* > *Akh-RNAi*, *Dar-2-RNAi*). Increased starvation resistance of *Akh* > *Dar-2-RNAi* animals compared to the control indicates that knock down of *Dar-2* in the APCs reduces AKH signaling. Ctrl: *UAS-Dicer2*, *UAS-Dar-2-RNAi/+*. All RNAi flies were significantly starvation resistant compared to the control in all starvation experiments (logrank test: *P* < 0.0001). Likewise *Akh* > *Akh* flies were significantly starvation-sensitive (logrank test: *P* < 0.0001). (**F**) Starvation resistance in flies with reduced *Dar-2* expression in the IPCs (*dilp2* > *Dar-2-RNAi*) or the DAR-2 producing cells (*Dar-2* > *Dar-2-RNAi*). Increased starvation resistance of *dilp2* > *Dar-2-RNAi* indicates reduced insulin signaling in these animals. Together, this suggests that AstA is a positive regulator of both AKH and DILP. Consistently, starvation resistance was also increased in *Dar-2* > *Dar-2-RNAi* animals with reduced *Dar-2* expression in cells expressing the receptor, including the APCs and IPCs. *UAS-Dicer2* was included since efficient neuronal knock down often requires overexpression of *Dicer2*. Ctrl: *UAS-Dicer2*, *UAS-Dar-2-RNAi/+.* All RNAi flies were significantly starvation resistant compared to the wild type control in all starvation experiments (logrank test: *P* < 0.0001). *: *P* < 0.05; **: *P* < 0.01; ***: *P* < 0.001 (Student’s t-test).

To investigate whether AstA regulates DILP and AKH signaling, expression of *TeTxLC.tnt*, the tetanus toxin light chain, which inhibits neurotransmitter exocytosis^28^, and *NaChBac*, a bacterial sodium channel that increases neuronal excitability^29^, was targeted to the AstA-producing neurons using *AstA-Gal4* (*AstA>*). Previously, it has been shown that expression of *NaChBac* using *AstA*>, which expresses Gal4 specifically in AstA-positive neurons, is sufficient to change the activity of the AstA neurons^18^. To examine the effects of AstA neuronal activity on APC and IPC activities, we measured changes in expression of *Akh*, *dilp2* and *dilp3*, and metabolic genes that are influenced by AKH and DILP signaling. Although the expression of *Akh* was not influenced in *AstA* > *TeTxLC.tnt* and *AstA* > *NaChBac* animals, the expression of the AKH receptor gene (*akhr*) was increased in *AstA* > *TeTxLC.tnt* animals where the activity of AstA-neurons is inhibited (Fig. 2A). Increased *akhr* expression may indicate an upregulation of the receptor in response to reduced levels of AKH in circulation. Moreover, activation of the AstA-neurons (*AstA* > *NaChBac*) induced *target of brain insulin (tobi)*, an α-glucosidase homolog which is stimulated by both AKH and the DILPs released from the IPCs^30^. Activation of the AstA neurons also promoted the expression of both *dilp2* and *dilp3* consistent with the increased expression of *tobi* (Fig. 2B). Although *dilp2* was also upregulated by inhibition of the AstA neurons (*AstA* > *TeTxLC.tnt*), a dramatic drop in *dilp3* expression was observed under these conditions. This suggests that AstA neuronal activity influences *dilp* expression in the IPCs, and especially *dilp3* seems to be tightly associated with the activity the AstA producing neurons. In comparison, the moderate upregulation of *dilp2* expression by both activation and inactivation of AstA neurons may indicate a more indirect and perhaps a compensatory regulation of *dilp2* expression by AstA neuronal activity. The transcription of *eIF4E-binding protein* (*4EBP*), encoding an inhibitor of translation which is suppressed by DILP signaling^31^,^32^, was not significantly changed.

To further characterize the phenotype associated with *AstA* loss of function, we generated a mutation in the *AstA* locus using the CRISPR/Cas9 system^33^. We recovered a line carrying a mutation that deletes part of the *AstA* gene including the start codon, which we named *AstA*^*SK1*^ (Fig. S2f). Since ablation of either the APCs or the IPCs results in increased starvation resistance^3^,^6^,^12^, we examined the resistance of *AstA*^*SK1*^ mutant flies to starvation. Flies with *AstA*^*SK1*^ mutation survive significantly longer under starvation compared to the controls (Fig. 2C). We used *y*^*2*^ *cho*^*2*^ *v*^*1*^ and *y*^*1*^ *w*^*1118*^ and a cross between these two lines as isogenic controls, since the mutation was generated in these genetic backgrounds. To determine whether *AstA*^*SK1*^ is a strong loss of function allele and ensure that the phenotype is caused by a specific mutation in the *AstA* locus, we tested the *AstA*^*SK1*^ allele over the *Df(3R)BCS519* deficiency that covers the *AstA* locus. *AstA*^*SK1*^/*Df(3R)BCS519* flies are starvation resistant, like *AstA*^*SK1*^ homozygous flies indicating that *AstA*^*SK1*^ is a strong loss of function mutation. Since *tobi* is a target of both DILP and AKH signaling and that its expression was altered by AstA neuron activation (Fig. 2A), we analyzed the expression of *tobi* in flies lacking *AstA*. We found that *AstA*^*SK1*^ homozygous flies and *AstA*^*SK1*^/*Df(3R)BCS519* flies exhibit a strong reduction in expression of *tobi*, similar to the decrease in *tobi* expression observed in *Akh*^*SK1*^ animals with a mutation in the *Akh* gene that disrupts the *Akh* reading frame (Fig. 2D, Fig. S2f). Like *AstA*^*SK1*^ mutants, the *Akh*^*SK1*^ mutant flies are also starvation resistant (data not shown) as previously reported for animals that lack *Akh* due to genetic ablation of the APCs^3^. Taken together this indicates a complex interplay between AstA, DILP and AKH signaling.

AstA is expressed both in the nervous system (Fig. S2d) and in endocrine gut cells^18^ and its release from these different subgroups of cells may be temporally distinct during natural feeding conditions. These different sources of AstA may affect DILP and AKH signaling independently. To determine whether the changes in expression of metabolic genes regulated by AKH and DILP signaling is caused by a direct effect of AstA on the IPCs, APCs or other metabolic changes that has an indirect effect, we analyzed the consequence of reducing *Dar-2* expression in these cells on the survival under starvation. Using an RNAi line that efficiently reduces expression of *Dar-2* (Fig. S1d and^23^), we silenced *Dar-2* in either the IPCs (*dilp2* > *Dar-2-RNAi*) or APCs (*Akh* > *Dar-2-RNAi*). Knock down of *Dar-2* in the APCs increased resistance to starvation significantly compared to the control, indicating that these animals have reduced AKH signaling (Fig. 2E). On the other hand, overexpression of *Akh* in the APCs made animals significantly more starvation sensitive compared to the control suggesting that these animals release more AKH into circulation which promotes energy mobilization. The starvation resistance observed after knock down of *Dar-2* in the APCs was not reversed by *Akh* overexpression indicating that the increased *Akh* transcription does not increase AKH release. This indicates that DAR-2 is important for the release of AKH consistent with the data showing that changing the activity of the AstA producing neurons does not influence *akh* expression. Reduction of *Akh* expression in the APCs made animals more resistant to starvation than when expression of *Dar-2* was reduced in these cells. However, when *Akh* and *Dar-2* expression was reduced simultaneously, the flies became even more starvation resistant than when these genes were knocked down individually. This suggests a genetic interaction between *Dar-2* and *Akh* that supports DAR-2 modulation of AKH release.

Interestingly, reducing the expression of *Dar-2* in the IPCs (*dilp2* > *Dar-2-RNAi*) also increased starvation resistance compared to the control (Fig. 2F). This suggests that *dilp2* > *Dar-2-RNAi* flies may have reduced insulin signaling, indicating that AstA is important for both AKH and DILP release. Consistent with these observations, knock down of the receptor in cells expressing *Dar-2* (*Dar-2* > *Dar-2-RNAi*) also increased survival time significantly under starvation. Although *Dar-2* > *Dar-2-RNAi* animals survive longer under starvation compared to the control, they are less resistant to starvation compared to *dilp2* > *Dar-2-RNAi* flies, which may be due to different strengths of the *Dar-2>* versus *dilp2>* drivers. Another possibility is that simultaneous silencing of *Dar-2* in both the CC and IPCs using the *Dar-2*> may result in another AKH to DILP ratio than when silencing *Dar-2* only in the IPCs using *dilp2>*, which may have a different effect on starvation survival. Together these observations suggest that AstA may be involved in energy mobilization through regulation of circulating AKH and DILP levels.

### Reducing *Dar-2* mRNA levels in the APCs and IPCs affects metabolic target genes

**Figure 3:**
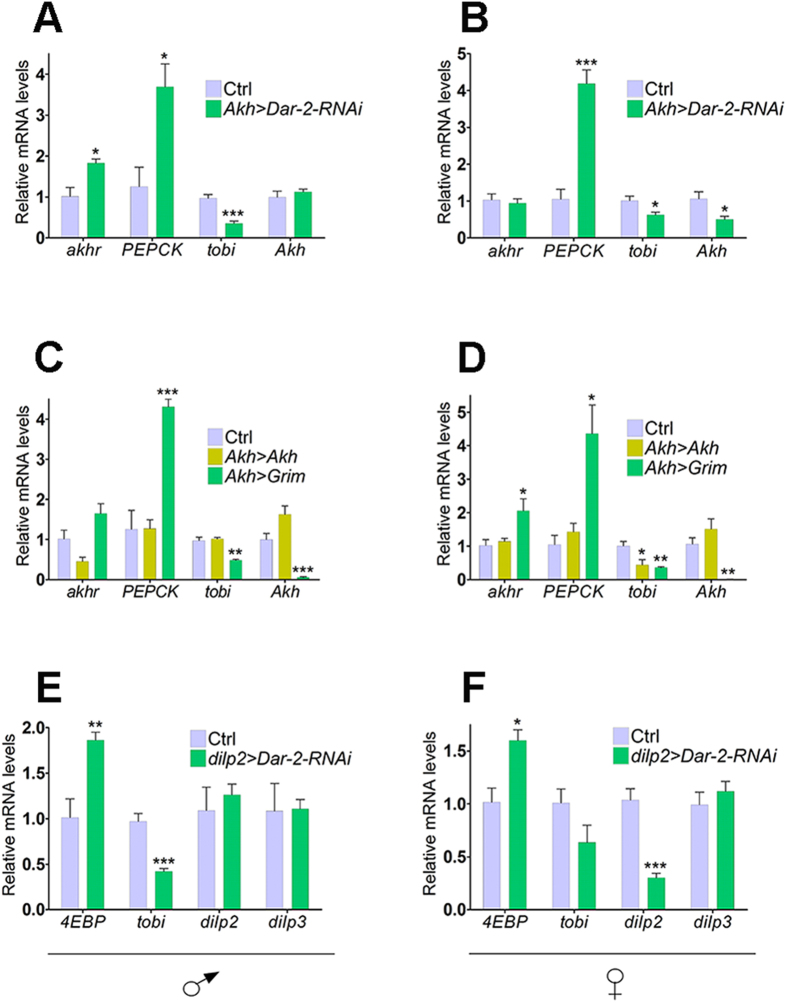
Reducing *Dar-2* mRNA levels in the APCs or IPCs affects expression of key metabolic genes. (**A**,**B**) Expression of genes related to AKH signaling in males (**A**) and females (**B**) with reduced *Dar-2* expression in the APCs (*Akh* > *Dar-2-RNAi*). Changes in AKH and metabolic gene expression in *Akh* > *Dar-2-RNAi* animals shows that AstA regulates AKH signaling. (**C**,**D**) Effects on expression of genes related to AKH signaling by increasing (*Akh* > *Akh*) or decreasing *(Akh* > *Grim*: APC-ablated) AKH levels in males (**C**) and females (**D**). Alterations observed in *Akh* > *Dar-2-RNAi* animals mimic those in APC-ablated flies suggesting that the effect of AstA on AKH signaling is stimulatory. (**E**,**F**) Expression of genes related to DILP signaling in males (**E**) and females (**F**) with reduced *Dar-2* expression in the IPCs (*dilp2* > *Dar-2-RNAi*). Increased expression of *4EBP* and decreased expression of *tobi* in *dilp2* > *Dar-2-RNAi* animals indicate a reduction in systemic insulin suggesting that AstA is a positive regulator of DILP signaling. *UAS-Dicer2* was included to enhance the RNAi effect. Ctrl: *UAS-Dicer2*, *UAS-Dar-2-RNAi*/+. Error bars indicate standard errors (n = 4). *: *P* < 0.05; **: *P* < 0.01; ***: *P* < 0.001 (Student’s t-test).

To further evaluate the effects of AstA on AKH and DILP signaling, we measured how cell specific *Dar-2-RNAi* affected transcript levels of key metabolic target genes influenced by AKH or DILP signaling. To investigate differences between the sexes, both males and females were analyzed. In male flies, reducing the expression of *Dar-2* in the APCs did not affect expression of *Akh* consistent with the notion that AstA is involved primarily in the regulation release rather than expression of *Akh* from the CC (Fig. 3A). On the other end, expression of *tobi* has been shown to be dependent of AKH release from the CC^30^. Consistent with a reduction in AKH release in *Akh* > *Dar-2-RNAi* males, we observed a decrease in *tobi* mRNA combined with an increase of *akhr* mRNA. Furthermore, a strong increase in expression of *phosphoenolpyruvate carboxykinase* (*PEPCK*), coding for a key enzyme in the gluconeogenesis^34^, supports that glycogen breakdown, a process promoted by AKH, is impaired consistent with reduced AKH signaling and a compensatory increase in gluconeogenesis. Similar effects on the expression of *PEPCK* and *tobi* were observed in females, but *Akh* expression was also reduced (Fig. 3B).

Next, we analyzed expression of these AKH signaling target genes in flies where *Akh* was either overexpressed or depleted by genetic ablation (*Akh* > *Grim*) of the APCs^35^. Consistent with previous observations, we found a reduction in *tobi* expression in APC-ablated animals^30^, like observed in *Akh*^*SK1*^ mutants (Fig. 1D). Alterations in expression of the AKH-influenced metabolic genes observed in animals with reduced *Dar-2* expression in the APCs mostly mimic those seen in APC-ablated animals and not flies overexpressing *Akh* (Fig. 3A–D). Furthermore, we found that transcription of the *brummer* (*bmm*) lipase is induced 6 hr after starvation in *Akh* > *Dar-2-RNAi* (Fig. S3e), like observed for animals lacking AKH or its receptor^7^. Together this indicates that lack of DAR2 in the APCs reduces AKH signaling, implying that AstA has a positive effect on AKH signaling.

Silencing of the expression of *Dar-2* in the IPCs reduced *dilp2* expression in females, but not in males (Fig. 3E,F). Although expression of *dilp2* and *dilp3* was not affected in *dilp2* > *Dar-2-RNAi* males, increased the expression of *4EBP* was observed in both sexes. Expression of *4EBP*, a direct target of FOXO, is upregulated in response to repressed insulin signaling^31^,^32^ and can be used as a proxy for global insulin signaling^36^,^37^. Expression of *tobi* is dependent not only on AKH, but also DILP signaling, which means that animals with reduced IPC release of DILPs have lower *tobi* transcript levels^30^. Consistent with the increase in *4EBP* in *dilp2* > *Dar-2-RNAi* animals, we observed that IPC-specific loss of *Dar-2* was associated with a reduction in *tobi* expression. This indicates that silencing of *Dar-2* in the IPCs causes a drop in systemic DILP signaling. Together these data suggest that AstA acts directly on the IPCs and APCs and has a positive influence on DILP and AKH signaling.

### DAR-2 is involved in the coordination of AKH and DILP signaling

**Figure 4:**
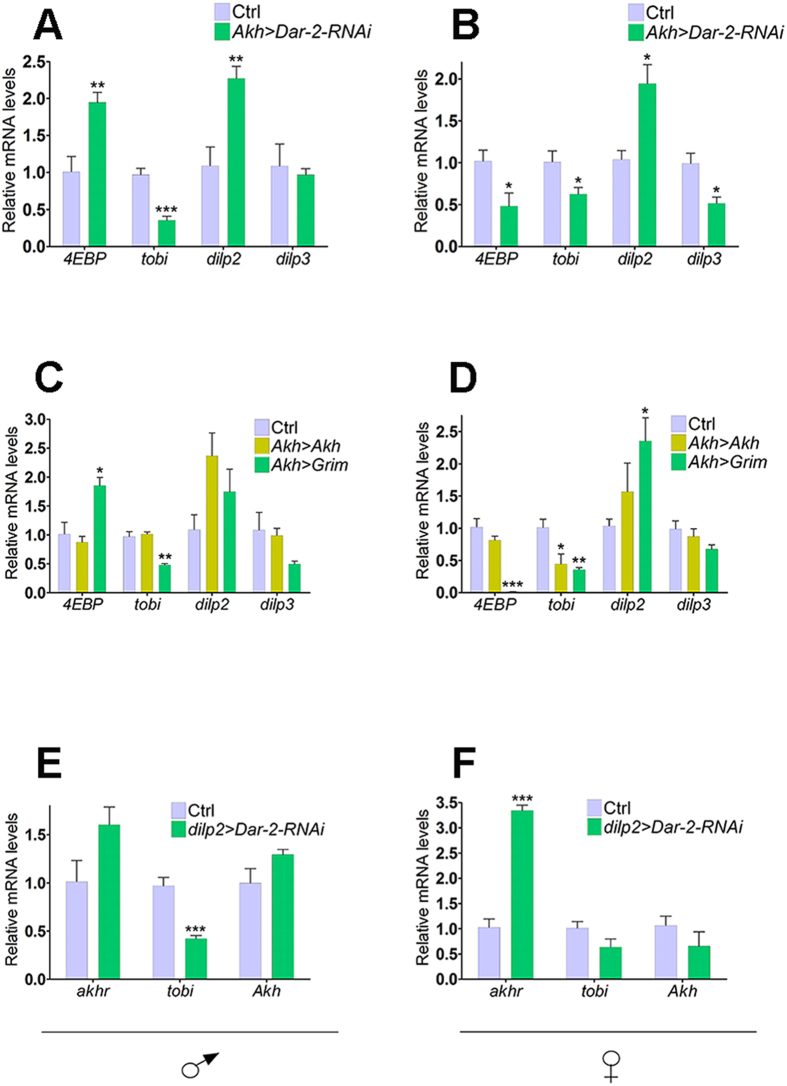
DAR-2 influences the interactions between AKH and DILP signaling. (**A**,**B**) Expression of genes related to DILP signaling in males (**A**) and females (**B**) with reduced *Dar-2* expression in the APCs (*Akh* > *Dar-2-RNAi*). Changes in *dilp2* and *-3* expression in *Akh* > *Dar-2-RNAi* animals suggest an interaction between AstA-regulated signaling from the APCs and DILP signaling that is sexually dimorphic based on the opposing effects on *4EBP* in males and females (**C**,**D**) Effects on expression of genes related to DILP signaling by increasing (*Akh* > *Akh*) or decreasing (*Akh* > *Grim*) AKH levels in males (**C**) and females (**D**) supports a sexually dimorphic interaction between AKH and DILP signaling. (**E**,**F**) Expression of genes related to AKH signaling in males (**A**) and females (**B**) with reduced *Dar-2* expression in the IPCs (*dilp2* > *Dar-2-RNAi*). Increased *akhr* expression in female *dilp2* > *Dar-2-RNAi* indicate that AstA-regulated DILP signaling directly or indirectly influences AKH signaling. *UAS-Dicer2* was included to enhance the RNAi effect. Ctrl: *UAS-Dicer2*, *UAS-Dar-2-RNAi*/+. Error bars indicate standard errors (n = 4). *: *P* < 0.05; **: *P* < 0.01; ***: *P* < 0.001 (Student’s t-test).

Since the balance between AKH and the DILPs is important for nutrient homeostasis and interactions between the APCs and the IPCs have been suggested^30^,^38^, we examined whether AstA is involved in crosstalk between these two cell types. To investigate if DILP signaling is regulated by signals from the APCs, and examine if this regulation is influenced by DAR-2, we analyzed the expression of *dilp2* and *−3* and their target genes in *Akh* > *Dar-2-RNAi* animals (Fig. 4A,B). Expression of *dilp2* was significantly increased in these animals. For *dilp3* expression, we observed a reduction in females but not in males. While the reduced *tobi* expression in *Akh* > *Dar-2-RNAi* animals may be a result of low AKH signaling, the effects on *4EBP* expression supports an effect on systemic DILP signaling, although it cannot be excluded that this is an indirect effect of changes in AKH signaling. Remarkably, we observed that *4EBP* expression was reduced in *Akh* > *Dar-2-RNAi* females, while it was increased in males, suggesting a sexually dimorphic interaction between AKH and DILP signaling. Changes in expression of genes associated with DILP signaling in the APC-ablated flies was similar to those seen in *Akh* > *Dar-2-RNAi* animals, including the sex-specific differences in *4EBP* expression and upregulation of *dilp2*. Together these results suggest that the APCs directly or indirectly play a role in regulation of systemic DILP signaling in a sex-specific manner (Fig. 4C,D). This also indicate that alterations in DILP signaling may indirectly contribute to the increased survival resistance associated with loss of *Dar-2* in the APCs or with silencing of *Akh* (Fig. 2E). We observed no effect of lack of *Akh* on *dilp3* mRNA levels. While our data are consistent with the findings of recent study^39^, an earlier study reported that loss of *Akh* induces *dilp3* expression^30^. We also confirmed that *dilp3* mRNA levels are unaffected in the *Akh* mutant (data not shown).

We also measured the expression of *Akh* and *akhr* in animals with reduced *Dar-2* expression in the IPCs, to examine if AstA regulation of the IPCs affects AKH signaling (Fig. 4E,F). Although a direct effect on *Akh* expression was not observed, *dilp2* > *Dar-2-RNAi* animals exhibited a more than three-fold increase in *akhr* expression in females. Since a reduction in AKH signaling in APC-ablated animals was associated with a compensatory increase in *akhr* expression (Fig. 3D), it is likely that AstA stimulated DILP signaling has a positive effect on AKH signaling. Another possibility is that DILP signaling regulates *akhr* transcription in the fat body, thereby affecting the response to AKH.

### Loss of AstA results in accumulation of lipids in the fat body

**Figure 5:**
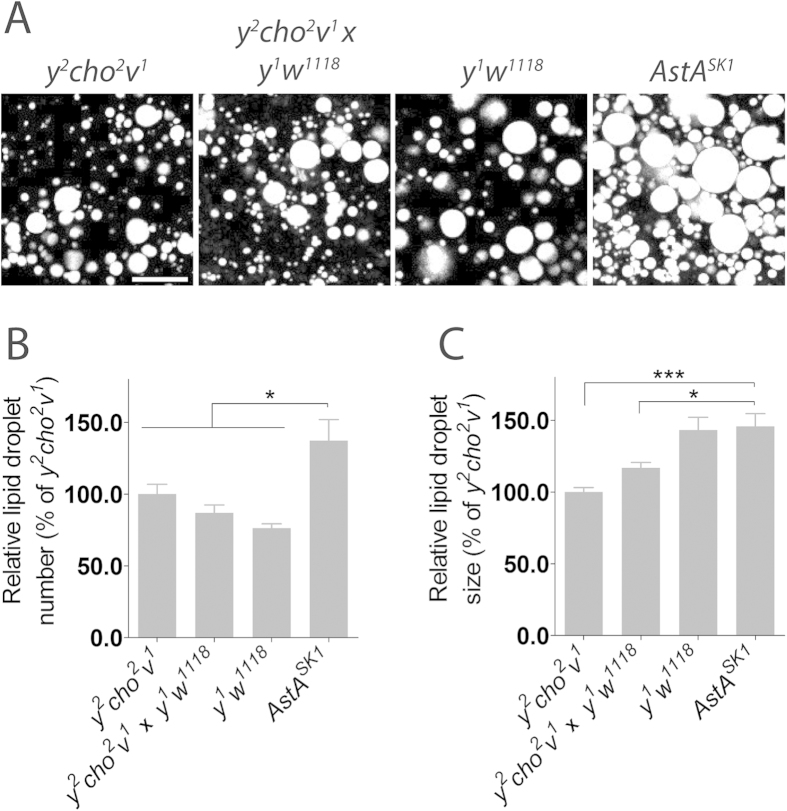
Effect of *AstA* loss of function on fat body lipid accumulation. (**A**) Lipid droplets were visualized in the fat body of adult males using CARS microscopy in animals lacking *AstA* (*AstA*^*SK1*^) and control animals (*y*^*2*^ *cho*^*2*^ *v*^*1*^, *y*^*1*^ *w*^*1118*^ and *y*^*2*^ *cho*^*2*^ *v*^*1*^ x *y*^*1*^ *w*^*1118*^: offspring from crossing these lines). (**B**,**C**) Quantification of lipid droplet number and size in adult fat body based on confocal images. The data shown represent the means and error bars indicate standard errors (n = 8). Scale bar, 10 μm. *: *P* < 0.05; ***: *P* < 0.001 (Student’s t-test).

In *Drosophila*, fat is stored in lipid droplets in the fat body, which is dependent both on AKH and DILP signaling^7^,^40^,^41^. *dilp* and *Akh* pathway mutants have elevated lipid levels and loss of AKH signaling and insulin resistance have been associated with increased fat body lipid droplet storage. If AstA affects metabolism, as suggested by its influence on AKH and DILP signaling and key metabolic genes, then loss of *AstA* may lead to a change in the abundance of lipid droplets in the fat body. To test this possibility, we examined lipid droplets in the adult fat body using Coherent Anti-Stokes Raman Scattering (CARS) microscopy, a label-free technique that allows visualization of lipids by imaging^42^. Loss of *AstA* increased the number of fat body lipid droplet compared to the controls (Fig. 5), indicating that lack of AstA is associated with accumulation of high fat body levels of lipids. This finding is consistent with the notion that AstA is involved in the control AKH and DILP release, since reduced AKH and DILP signaling promotes lipid accumulation in the fat body. This provides evidence indicating that *AstA* mutants have metabolic defects and excess energy reserves which is consistent with their increased resistance to starvation. In contrast, changing the activity of the AstA neurons was recently reported not to affect starvation resistance and lipid levels in *Drosophila*^18^. A likely explanation for the observed differences is that changing AstA neuron activity may produce another phenotype compared to a mutation that affects AstA signaling in all tissues, including the endocrine gut cells.

### AstA is affected differently by carbohydrate and protein rich diets and regulates preferences for these nutrients

**Figure 6:**
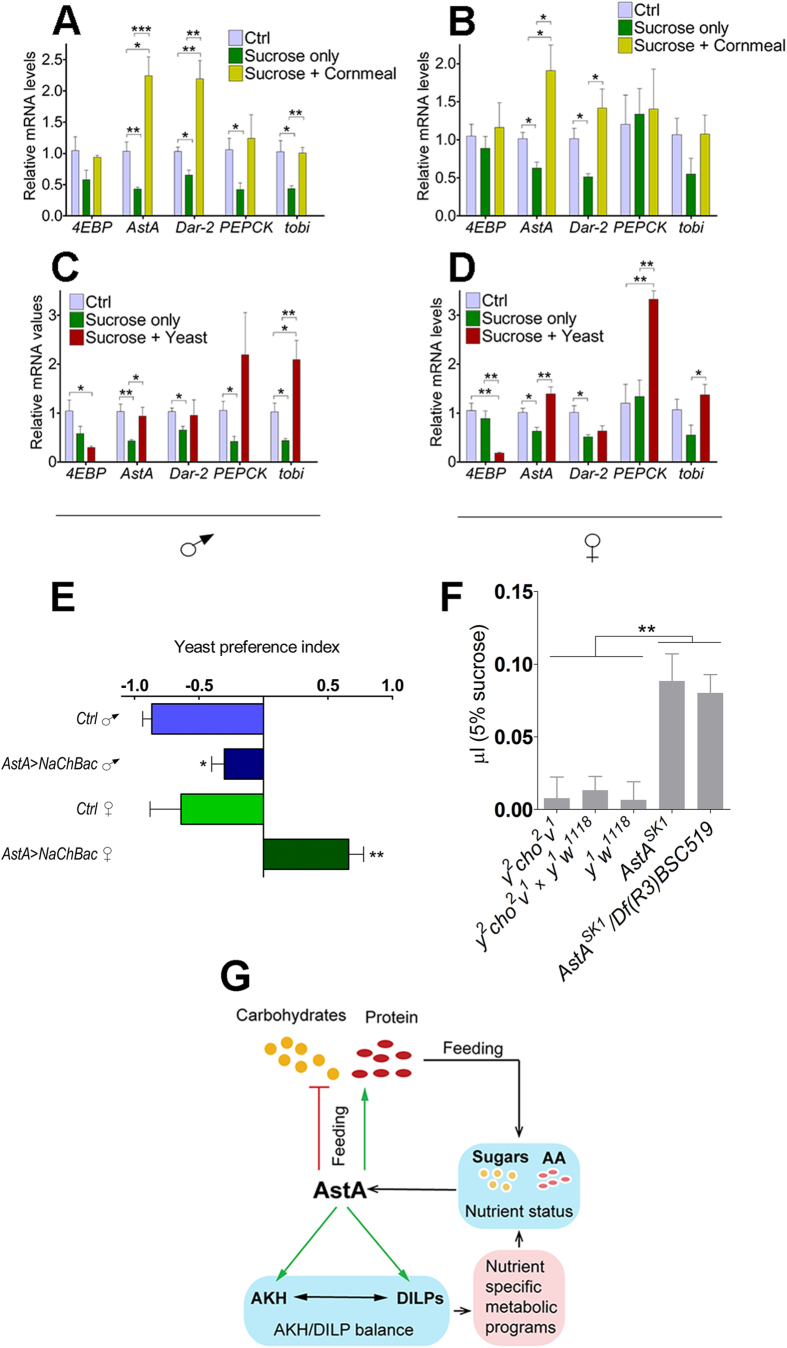
AstA is affected differentially by protein and sugar and regulates preference for these nutrients and food intake. (**A**–**D**) Expression of genes related to AstA signaling and metabolism in wild type (*w*^*1118*^) male (**A**,**C**) and female (**B**,**D**) flies fed on cornmeal (Ctrl), a restricted diet of 1% sucrose for 48 hours (Sucrose only) or re-fed for 24 hours after dietary restriction with either cornmeal (Sucrose + Cornmeal) (**A**,**B**) or yeast (Sucrose + Yeast) (**C**,**D**). After nutrient restriction, a carbohydrate rich cornmeal diet strongly induced *AstA* and *Dar-2* expression, while a protein rich yeast diet only had a moderate effect on expression of *AstA* with no effects of *Dar-2* transcripts. Error bars indicate standard errors (n = 4). (**E**) Yeast preference index indicating the preference for dietary protein. Males and females with increased (AstA > *NaChBac*) AstA neuron activation were tested in a two choice feeding assay for their preference for sucrose versus yeast (protein). Negative values indicate a preference for sucrose and positive values indicate a preference for yeast. Ad-libitum-fed *AstA* > *NaChBac* flies had a stronger preference for protein rich yeast compared to the control. Ctrl: *AstA-Gal4/+*. Error bars indicate standard errors (n = 3). (**F**) Sucrose consumption of adult males lacking *AstA* (*AstA*^*SK1*^ *and AstA*^*SK1*^*/Df(3R)BSC519*) compared to control animals (*y*^*2*^ *cho*^*2*^ *v*^*1*^, *y*^*1*^ *w*^*1118*^ and *y*^*2*^ *cho*^*2*^ *v*^*1*^ x *y*^*1*^ *w*^*1118*^: offspring from crossing these lines). Sucrose intake was measured using the Capillary Feeding (CAFE) assay. Error bars indicate standard errors (n = 10). *: *P* < 0.05; **: *P* < 0.01; ***: *P* < 0.001 (Student’s t-test). (**G**) Model: The proposed role of AstA in coordinating metabolism and feeding decision to balance nutrient intake according the internal nutrient state. AstA is affected differentially by dietary sugar and protein and guide metabolic programs, through the balance between AKH and DILPs, important to maintain homeostasis on food with different sugar:protein ratios. Activation of AstA neurons change the preference for protein rich food, showing that AstA plays a key role in the decision-making processes that assign value to dietary sugar and protein and guide food choice.

Since the interplay between the DILPs and AKH has previously been suggested to mediate metabolic programs related to carbohydrate versus protein rich diets^30^, we asked whether AstA signaling is regulated in response to feeding and the type of nutrients. We measured the expression of *AstA* and *Dar-2* and key metabolic target genes in animals fed on either carbohydrate rich (cornmeal) or protein rich (yeast) food after 48 hours on a restricted low nutrient diet (1% sucrose) and compared it to control flies kept on cornmeal. After nutrient restriction, gene expression of *AstA* and *Dar-2* was down regulated compared to the control and the expression of several metabolic genes was decreased in males indicating a general reduction of metabolism (Fig. 6A–D). When flies were fed on a high sugar diet after nutrient restriction, the expression of *AstA* and *Dar-2* exhibited a strong response and increased to levels above that of control flies (Fig. 6A,B), while expression of the metabolic target genes only rebounded to levels similar to the control.

Feeding on a protein rich diet also increased *AstA* expression, but not as strongly as the high sugar diet, and *Dar-2* expression did not increase (Fig. 6C,D). The type of diet, protein versus carbohydrate, also affected expression of the metabolic genes differentially. While the carbohydrate rich meal increased expression of *4EBP*, the protein rich diet further reduced expression compared to the levels observed in nutrient restricted animals. The drop in *4EBP* expression on the protein rich yeast diet indicates that dietary protein strongly increases systemic DILP signaling. Levels of *tobi* and *PEPCK* mRNA also increased more on the protein rich yeast compared to the carbohydrate rich cornmeal, suggesting an increase in glycogen breakdown and gluconeogenesis, processes expected to be important on a dietary source low on carbohydrates. The increase in DILP signaling as measured by changes in *4EBP* and *tobi* expression on a protein rich diet is consistent with previous observations^30^,^43^. Taken together this shows that *AstA* and *Dar-2* expression is regulated by nutrient intake and that the response depends on the carbohydrate and protein composition of the diet.

The differential response in *AstA* expression to carbohydrates and proteins prompted us to investigate whether AstA signaling is involved in feeding decisions related to intake of these different types of nutrients. To test if AstA affects the preference between sugar and proteins, we performed a two-choice feeding assay^44^, where flies were allowed to choose between a sucrose medium colored with a red dye and a yeast medium colored with a blue dye. We observed that ad-libitum-fed control flies kept on standard food conditions prefer sucrose (Fig. 6E) consistent with previous observations^44^. In contrast, ad libitum fed *AstA* > *NaChBac* flies with activation of the AstA neurons showed a stronger preference for the protein rich yeast diet compared to control flies. We also found a stronger preference for yeast in females compared to males. These data suggest that AstA is involved in assigning nutritional value to these dietary components and that increased AstA signaling may mimic sugar satiety or perhaps reflect a protein-hunger driven behavior. We therefore asked whether reducing AstA signaling promotes motivation to consume dietary sugar. To test this possibility, we measured sucrose feeding in the *AstA*^*SK1*^ mutants. We found that loss of *AstA* enhances sucrose intake compared to the control flies (Fig. 6F). These results also agree with a recent report showing that inhibition of the AstA neurons enhances the acceptance of sugar in an otherwise unpalatable food resource^18^. Altogether our data suggest that AstA respond to changes in nutrient intake and plays an important role in controlling metabolism and feeding (Fig. 6G). This suggests that AstA may be involved in a nutrient sensing mechanism that assign nutritional value to sugar and protein and guide feeding decisions to maintain the balance between energy requirements and food intake.

## Discussion

In order to adjust energy homeostasis to different environmental conditions, feeding-related behavior needs to be coordinated with nutrient sensing and metabolism. Our data suggest that AstA is a modulator of AKH and DILP signaling that control metabolism and nutrient storage, but also affects feeding decisions. The positive effect of AstA on AKH signaling indicated by our observations is supported by the recent finding that expression of a presumably constitutive active mu opioid receptor, a mammalian GPCR which is also closely related to DAR-2, stimulates AKH release from the APCs in *Drosophila*^45^. Moreover, AstA-type peptides have also been shown to stimulate AKH release in *Locusta migratoria*^46^. AKH is primarily regulated at the level of secretion to allow a rapid response to metabolic needs^47^. Considering that we only observed a minor effect of *Dar-2* silencing in the APCs on *Akh* transcription, it is likely that AstA primarily acts at the level of AKH release in *Drosophila*.

Our data suggest regulation of both the DILPs and AKH by AstA indicating a close coupling between the activity of these two hormones. Consistent with this notion, our results also indicate a feedback relationship between the IPCs and APCs. The IPCs have processes that contact the CC cells^13^,^14^ and it is possible that DILPs released from these affect AKH release. Our findings are supported by a previous study that identifies a tight association between DILPs and AKH secretion in *Drosophila*^30^. Furthermore, it was recently found that AKH regulates DILP3 release from the IPCs, and that sugar promotes DILP3 release, while DILP2 release is amino acid dependent^39^. Interestingly, our data, which suggest that AstA is involved in the cross-talk between DILPs and AKH related specifically to sugar and protein, also indicate that AstA has a strong influence on *dilp3* expression. Why is the relationship between the DILPs and AKH so tight? Even though insulin-like peptides reduce hemolymph sugar, they also reduce the content of stored glycogen and lipids^11^,^12^, like AKH^3^,^6^. Consistent with this, both AKH and the DILPs stimulate expression of *tobi*, which encodes a glycosidase believed to be involved in glycogen breakdown^30^. However, since AKH and the DILPs have opposing effect on hemolymph sugar levels, a balance between these hormones is presumably required to maintain homeostasis. It is likely that different sources of AstA affect these two hormones, since the IPCs are located in the brain in proximity of AstA-positive neurites, while AstA-positive processes do not innervate the CC^48^. Thus, it is likely that neuronal-derived AstA affects DILP secretion from the IPCs, while circulating AstA, which may be released from the endocrine cells of the gut, may be the source of AstA that acts on the APCs to regulate AKH. AstA regulation of DILP and AKH release may therefore not occur simultaneously and could also depend on the type of nutrient ingested, or be sequential. Since our data suggest feedback regulation between AKH and DILP, the overall outcome of simultaneous AstA induced activation of both cell types will not necessarily be a strong and equal increase in both hormones in the hemolymph. It is possible that AstA is involved in metabolic balancing, adjusting the ratio between AKH and DILPs in response to different dietary conditions. In mammals, glucagon and insulin are secreted simultaneously when the animal feeds on a protein-rich diet, to prevent hypoglycemia and promote cellular protein synthesis, since insulin is strongly induced after ingestion of amino acids^49^,^50^,^51^. A similar mechanism has been proposed to explain the relationship between DILPs and AKH in *Drosophila*^30^. The balance between DILP and AKH therefore may be important for resource allocation into growth and reproduction.

Several differences in the expression of genes involved in energy mobilization were observed between males and females, which possibly reflects sex-specific strategies for energy mobilization and allocation of resources towards reproduction. Interestingly, *4EBP* expression was significantly decreased in females with reduced AKH signaling, but upregulated in males. This suggests that in females AKH has a strong negative influence on DILP signaling that is not present in males. Why does the interaction between AKH and DILPs differ between sexes? An interesting possibility is that this sexually dimorphic interaction is related to the different preferences and requirements for sugar and protein in males and females. Males generally have a higher preference for sugar compared to females that prefer more dietary protein and show strong correlation between amino acid uptake, insulin and reproduction^44^,^52^,^53^. In both mammals and *Drosophila* the balance between insulin and glucagon/AKH is important for nutrient homeostasis in response to high-protein versus high-sugar diets^30^,^50^,^51^. This balance ensures that insulin promotes protein synthesis in response to dietary amino acids, while maintaining sugar levels stable, a function possibly important in females to allocate the high consumption of amino acids into reproduction. Thus, the sex-specific interplay between DILPs and AKH likely reflects difference in the metabolic wiring of males and females that underlie the sexually dimorphic reproductive requirements for dietary sugar and protein.

Interestingly, *AstA* expression showed a general increase after feeding with a stronger transcriptional response of both *AstA* and *Dar-2* to the carbohydrate rich diet compared to the protein rich diet. AstA may therefore be important for coordinating carbohydrate and protein dependent metabolic programs. The strong response to carbohydrates indicates that AstA may be involved in signaling related to carbohydrate feeding, although increased transcription may not necessarily result in elevated release of the mature AstA peptide. Nonetheless, the data indicate that feeding regulates AstA-signaling and that the response is influenced by the food composition. Consistent with the notion that AstA is involved in different responses to dietary carbohydrate and protein, we found that flies with increased AstA neuronal activity increase their protein preference on the expense of their natural preference for sucrose. The AstA regulated circuitry may therefore be important for guiding the decision to feed on protein or sugar, a decision influenced by metabolic needs^44^. The AstA neurons have projections that may contact the Gr5a sugar sensing neurons and *AstA* > *NaChBac* flies with increased activity of the AstA neurons display reduced feeding and responsiveness to sucrose under starvation^18^. Thus, the increased preference for dietary protein in *AstA* > *NaChBac* flies observed here may be caused by reduced sucrose responsiveness. If AstA signaling is high after feeding on carbohydrates as indicated by our data showing increased expression of *AstA* and *Dar-2*, then an increase in AstA signaling might mimic carbohydrate satiety. In line with this view, our data show that animals lacking *AstA* enhance their intake of dietary sugar. AstA signaling may therefore increase the animals preference for essential amino acids, as suggested by a recent study indicating that amino acid depleted flies increased their taste sensitivity for amino acids, even when they were replete with glucose^54^. Based on our data, we therefore propose that AstA plays a central role in a circuitry important for encoding nutritional value related to these distinct nutrients and the regulation of feeding decisions and metabolic programs. Excess dietary sugar is associated with obesity^37^,^41^ and we find that flies lacking *AstA* enhance intake of sugar and have increased lipid storage droplets in their fat bodies, like animals lacking AKH or its receptor^7^. Thus, our data implicate AstA in regulation of appetite and food intake related to sugar, which is relevant for understanding obesity.

Our study suggests that AstA affects metabolism through its action on two key players, the DILPs and AKH. *AstA* expression is induced by feeding, but exhibits a differential nutritional response to dietary sugar and protein and influence metabolic programs and feeding choices associated with the intake of these nutrients. Interestingly, the homolog of AstA, galanin, regulates both feeding and metabolism in mammals and in *Caenorhabditis elegans* loss of the Allatostatin/galanin-like receptor *npr9* affects foraging behavior and nutrient storage^55^. Altogether our data suggest that AstA is part of a conserved mechanism involved in coordinating nutrient sensing, feeding decisions and metabolism to ensure adequate intake of amino acids and sugar to maintain nutrient homeostasis under different feeding conditions.

## Methods

### *Drosophila* stocks and mutants

The following lines of *Drosophila* were used; *w*^*1118*^ (Vienna *Drosophila* RNAi center: VDRC), *UAS-CD8-GFP* (Bloomington *Drosophila* stock center: BDSC, 5137), *UAS-Dicer2* (M. B. O´Connor), *UAS-Grim* (M. B. O´Connor), *UAS-Dar-2-RNAi* (VDRC, 1327), *UAS-Akh-RNAi* (VDRC, 105063), *UAS-Akh* (BDSC, 27343), *Akh-Gal4* (BDSC, 25684), *dilp2-Gal4* (E. Rulifson), *Dar-2-Gal4* (BDSC, 46581), *AstA-Gal4* (D. J. Anderson^18^), *UAS-TeTxLC.tnt* (BDSC, 28838), *UAS-NaChBac-EGFP* (BDSC, 9467), *Df(3R)BSC519/TM6C Sb*^*1*^ *cu*^*1*^ (BDSC, 25023). A mutation was generated in the *AstA* and *Akh* loci using the CRISPR/Cas9 system as described^33^. The target guide RNA (gRNA) sequences GCAGTAGGAGGTGGGCGTGAAGG for *AstA* and GGCCAGCAGCATGAAGAGCACGG for *Akh*, including the underlined 3 bp Protospacer Adjacent Motif (PAM) sequence, were used to generate *U6-gRNA* vectors. Transgenic *U6-gRNA* flies were established and indel mutations in the *AstA* and *Akh* loci were recovered in offspring from *nos-Cas9* and *U6-gRNA* flies. For each gene, six to eight independent F1 males were mated to balancer females. After three days, the male was removed from the culture vial and its genomic DNA was extracted and used as a template for PCR amplification of the target region. The PCR product was directly sequenced to identify a potential heterozygous indel mutation. For *AstA*, four out of six F1 males examined carried a small indel mutation. For *Akh*, a small indel mutation was present in all of eight F1 males examined. The *AstA*^*SK1*^ mutation removes 19 nt of the *AstA* locus including the ATG start codon, while the *Akh*^*SK1*^ mutation introduces a frameshift downstream of the ATG start codon (Fig. S2f). As controls for the *AstA*^*SK1*^ and *Akh*^*SK1*^ mutants, crosses between the *y*^*2*^ *cho*^*2*^ *v*^*1*^ and *y*^*1*^ *w*^*1118*^ lines, as well as the individual lines, were used since mutations were introduced into these genetic backgrounds. The *y*^*2*^ *cho*^*2*^ *v*^*1*^ and *y*^*1*^ *w*^*1118*^ lines had been generated by crossing the *y*^*1*^ *v*^*1*^, *y*^*2*^ *cho*^*2*^, *y*^*1*^ and *w*^*1118*^ (BDSC). To generate a knock-in GAL4 reporter line for *Dar-2*, a T2A-GAL4^56^ transgene was integrated into the *Dar-2* locus by CRISPR-mediated homologous recombination. T2A-GAL4 was placed immediately downstream of the final coding codon of *Dar-2*, such that the engineered locus encodes a bicistronic transcript that produces DAR-2 and GAL4 proteins, thereby allowing GAL4 to be expressed exactly in the same spatiotemporal pattern as endogenous DAR-2. Details of the transgene cassette used in the targeting vector will be described elsewhere. All flies were reared under 25 °C and 60% humidity and with 12:12 h light-dark cycle on standard cornmeal unless otherwise stated. To ensure similar nutritional conditions, an equal number of adult flies were kept in each vial for all experiments.

#### Immunostaining

Tissues of *Drosophila* adults or 3^rd^ instar larvae was dissected, fixated and incubated at 4 °C with the primary antibody and at room temperature with the secondary antibody. Antibodies were diluted as follows: rabbit anti-AstA (1:4000, H. J. Agricola), rabbit anti-DAR-2 (1:1000, S. Tobe), rabbit anti-AKH (1:600, J. Park), rabbit anti-DILP2 (1:2000, J.A. Veenstra), mouse anti-GFP (1:200 or 1:1000, JL-8, Clontech), goat anti-rabbit Alexa-fluor 555 (1:200), goat anti-mouse Alexa-fluor 488 (1:200 or 1:1000). DAPI was used for nuclear staining and images were obtained using confocal microscopy.

#### Quantitative PCR

Total RNA was isolated using RNeasy Mini kit (Qiagen) and treated with RNase-Free DNase to avoid genomic DNA contamination. RNA was extracted from four pooled females or five pooled males. For isolation of total RNA from adult CC, RNA was extracted from 10 adult CC for each sample. For measuring dietary effects, male and female *w*^*1118*^ were separated after eclosion and kept on normal cornmeal for 3 to 6 days before they were transferred to a restricted diet of agar containing 1% sucrose^43^,^57^ for two days. Hereafter they were fed either pure yeast or normal cornmeal for 24 hours. Control flies were kept at standard cornmeal and collected at the same age. For all other experiments RNA was extracted from animals 6 days after eclosion. cDNA was synthesized from oligo(dT) primers using the Superscript III reverse transcriptase (Invitrogen). Levels of transcripts were quantified using quantitative real time PCR (qPCR) with SYBR green as the reporter. Primers are given in the Table S1 and qPCR conditions were: 95 °C 10 min followed by 45 cycles of 95 °C for 15 sec, 60 °C for 15 sec and 72 °C for 15 sec. Melting curve analysis was carried out for all PCR reactions to verify homogeneity of the PCR products. As an internal standard, expression of the ribosomal protein *rpL23* was used for normalization as described^58^. Relative quantification of transcript levels was determined using the comparative Ct-methods.

#### Starvation assay

Flies were kept on standard cornmeal on a density of 20 animals per vial for 4–7 days after eclosion before they were starved by transferring to vials containing agar (1%). Prism software (GraphPad Prism software, version 4.02, San Diego California USA) was used to examine the significance of the starvation survival using the Kaplan-Meier method and a Mantel-Haenszel logrank test.

#### Feeding assays

Two-choice feeding preference assay was performed as described^44^. In brief, flies were kept on standard medium for 3–5 days after eclosion before they were offered the choice between sucrose medium (100 mM) containing a red dye and a yeast medium (5%, rich in protein) with a blue dye for 3 hours. Flies were scored according to the color of their abdomen. The “yeast preference index” was calculated and each experiment was repeated 3 times and each included 13–23 flies.

Quantification of sucrose intake was determined using the Capillary Feeding (CAFE) assay as described^59^. Males were kept on standard medium for 3–5 days after eclosion and then transferred to vials with 1% agar (to allow access to water) and a glass capillary containing 5% sucrose and 0.6 g/l Allura Red AC dye (Sigma-Aldrich) for 2 hours at 25 °C and 60% humidity. Blank controls without flies were included in each experiment to determine evaporation.

#### Determination of lipid storage droplets using Coherent Anti-Stokes Raman Scattering (CARS) microscopy

Lipid droplets were determined in fat body from adult flies 4-5 days after eclosion using CARS microscopy. Fat bodies were dissected in PBS and immediately imaged using a Leica TCS SP8 microscope with a CARS laser picoEmerald (OPO, >600 mW at 780 nm to 940 nm, pulse width 5 to 6 ps, 80 MHz;Pump, >750 mW at 1064 nm, pulse length 7 ps, 80 MHz) and the LAS AF/X software. Lasers were adapted to the CH stretching vibrational range by tuning the pump beam to 816.4 nm while keeping the Stokes beam constant at 1,064.6 nm. The output of the lasers was set to 1.3 W and the scan speed was 400 Hz. Signals were collected from the epi-CARS (E-CARS) and epi-SHG (E-SHG) detectors. Quantification of lipid droplet number and size was done using ImageJ (NIH).

## Additional Information

**How to cite this article**: Hentze, J. L. *et al.* The Neuropeptide Allatostatin A Regulates Metabolism and Feeding Decisions in *Drosophila*. *Sci. Rep.* **5**, 11680; doi: 10.1038/srep11680 (2015).

## Supplementary Material

Supplementary Information

## References

[b1] Baker K. D. & Thummel C. S. Diabetic larvae and obese flies-emerging studies of metabolism in *Drosophila*. Cell Metab 6, 257–266 (2007). 17908555 10.1016/j.cmet.2007.09.002 PMC2231808

[b2] Melcher C., Bader R. & Pankratz M. J. Amino acids, taste circuits, and feeding behavior in *Drosophila*: towards understanding the psychology of feeding in flies and man. J Endocrinol 192, 467–472 (2007). 17332516 10.1677/JOE-06-0066

[b3] Lee G. & Park J. H. Hemolymph sugar homeostasis and starvation-induced hyperactivity affected by genetic manipulations of the adipokinetic hormone-encoding gene in *Drosophila melanogaster*. Genetics 167, 311–323 (2004). 15166157 10.1534/genetics.167.1.311 PMC1470856

[b4] Kim S. K. & Rulifson E. J. Conserved mechanisms of glucose sensing and regulation by *Drosophila* corpora cardiaca cells. Nature 431, 316–320 (2004). 15372035 10.1038/nature02897

[b5] Isabel G., Martin J. R., Chidami S., Veenstra J. A. & Rosay P. AKH-producing neuroendocrine cell ablation decreases trehalose and induces behavioral changes in *Drosophila*. Am J Physiol - Regul Integr Comp Physiol 288, R531–538 (2005). 15374818 10.1152/ajpregu.00158.2004

[b6] Bharucha K. N., Tarr P. & Zipursky S. L. A glucagon-like endocrine pathway in *Drosophila* modulates both lipid and carbohydrate homeostasis. J Exp Biol 211, 3103–3110 (2008). 18805809 10.1242/jeb.016451 PMC2714167

[b7] Gronke S. *et al.* Dual lipolytic control of body fat storage and mobilization in *Drosophila*. PLoS Biol 5, e137 (2007). 17488184 10.1371/journal.pbio.0050137 PMC1865564

[b8] Brogiolo W. *et al.* An evolutionarily conserved function of the *Drosophila* insulin receptor and insulin-like peptides in growth control. Curr Biol 11, 213–221 (2001). 11250149 10.1016/s0960-9822(01)00068-9

[b9] Teleman A. A. Molecular mechanisms of metabolic regulation by insulin in *Drosophila*. Biochem J 425, 13–26 (2010). 20001959 10.1042/BJ20091181

[b10] Mirth C. K. & Riddiford L. M. Size assessment and growth control: how adult size is determined in insects. Bioessays 29, 344–355 (2007). 17373657 10.1002/bies.20552

[b11] Ceddia R. B., Bikopoulos G. J., Hilliker A. J. & Sweeney G. Insulin stimulates glucose metabolism via the pentose phosphate pathway in *Drosophila* Kc cells. FEBS letters 555, 307–310 (2003). 14644433 10.1016/s0014-5793(03)01261-4

[b12] Broughton S. J. *et al.* Longer lifespan, altered metabolism, and stress resistance in *Drosophila* from ablation of cells making insulin-like ligands. Proc Natl Acad Sci USA 102, 3105–3110 (2005). 15708981 10.1073/pnas.0405775102 PMC549445

[b13] Rulifson E. J., Kim S. K. & Nusse R. Ablation of insulin-producing neurons in flies: growth and diabetic phenotypes. Science 296, 1118–1120 (2002). 12004130 10.1126/science.1070058

[b14] Ikeya T., Galic M., Belawat P., Nairz K. & Hafen E. Nutrient-dependent expression of insulin-like peptides from neuroendocrine cells in the CNS contributes to growth regulation in *Drosophila*. Curr Biol 12, 1293–1300 (2002). 12176357 10.1016/s0960-9822(02)01043-6

[b15] Crown A., Clifton D. K. & Steiner R. A. Neuropeptide signaling in the integration of metabolism and reproduction. Neuroendocrinology 86, 175–182 (2007). 17898535 10.1159/000109095

[b16] Nassel D. R. & Winther A. M. *Drosophila* neuropeptides in regulation of physiology and behavior. Prog Neurobiol 92, 42–104 (2010). 20447440 10.1016/j.pneurobio.2010.04.010

[b17] Wang C., Chin-Sang I. & Bendena W. G. The FGLamide-allatostatins influence foraging behavior in *Drosophila melanogaster*. PloS one 7, e36059 (2012). 22558326 10.1371/journal.pone.0036059 PMC3338617

[b18] Hergarden A. C., Tayler T. D. & Anderson D. J. Allatostatin-A neurons inhibit feeding behavior in adult *Drosophila*. Proc Natl Acad Sci USA 109, 3967–3972 (2012). 22345563 10.1073/pnas.1200778109 PMC3309792

[b19] Lenz C., Williamson M. & Grimmelikhuijzen C. J. Molecular cloning and genomic organization of an allatostatin preprohormone from *Drosophila melanogaster*. Biochem Biophys Res Commun 273, 1126–1131 (2000). 10891383 10.1006/bbrc.2000.3062

[b20] Lenz C., Williamson M., Hansen G. N. & Grimmelikhuijzen C. J. Identification of four *Drosophila* allatostatins as the cognate ligands for the *Drosophila* orphan receptor DAR-2. Biochem Biophys Res Commun 286, 1117–1122 (2001). 11527415 10.1006/bbrc.2001.5475

[b21] Larsen M. J. *et al.* Type A allatostatins from *Drosophila melanogaster* and *Diplotera puncata* activate two *Drosophila* allatostatin receptors, DAR-1 and DAR-2, expressed in CHO cells. Biochem Biophys Res Commun 286, 895–901 (2001). 11527383 10.1006/bbrc.2001.5476

[b22] Woodhead A. P., Stay B., Seidel S. L., Khan M. A. & Tobe S. S. Primary structure of four allatostatins: neuropeptide inhibitors of juvenile hormone synthesis. Proc Natl Acad Sci USA 86, 5997–6001 (1989). 2762309 10.1073/pnas.86.15.5997 PMC297759

[b23] Wang C., Zhang J., Tobe S. S. & Bendena W. G. Defining the contribution of select neuropeptides and their receptors in regulating sesquiterpenoid biosynthesis by *Drosophila melanogaster* ring gland/corpus allatum through RNAi analysis. Gen Comp Endocrinol 176, 347–353 (2012). 22245290 10.1016/j.ygcen.2011.12.039

[b24] Lenz C., Williamson M. & Grimmelikhuijzen C. J. Molecular cloning and genomic organization of a second probable allatostatin receptor from *Drosophila melanogaster*. Biochem Biophys Res Commun 273, 571–577 (2000). 10873647 10.1006/bbrc.2000.2964

[b25] Birgul N., Weise C., Kreienkamp H. J. & Richter D. Reverse physiology in *Drosophila*: identification of a novel allatostatin-like neuropeptide and its cognate receptor structurally related to the mammalian somatostatin/galanin/opioid receptor family. EMBO J 18, 5892–5900 (1999). 10545101 10.1093/emboj/18.21.5892 PMC1171655

[b26] Fang P. H. *et al.* Central nervous system regulation of food intake and energy expenditure: role of galanin-mediated feeding behavior. Neurosci Bull 27, 407–412 (2011). 22108817 10.1007/s12264-011-1841-7 PMC5560389

[b27] Bowser P. R. & Tobe S. S. Immunocytochemical analysis of putative allatostatin receptor (DAR-2) distribution in the CNS of larval *Drosophila melanogaster*. Peptides 26, 81–87 (2005). 15626507 10.1016/j.peptides.2004.08.026

[b28] Sweeney S. T., Broadie K., Keane J., Niemann H. & O’Kane C. J. Targeted expression of tetanus toxin light chain in *Drosophila* specifically eliminates synaptic transmission and causes behavioral defects. Neuron 14, 341–351 (1995). 7857643 10.1016/0896-6273(95)90290-2

[b29] Yuan Q. *et al.* Light-induced structural and functional plasticity in *Drosophila* larval visual system. Science 333, 1458–1462 (2011). 21903815 10.1126/science.1207121 PMC4114502

[b30] Buch S., Melcher C., Bauer M., Katzenberger J. & Pankratz M. J. Opposing effects of dietary protein and sugar regulate a transcriptional target of *Drosophila* insulin-like peptide signaling. Cell Metab 7, 321–332 (2008). 18396138 10.1016/j.cmet.2008.02.012

[b31] Junger M. A. *et al.* The *Drosophila* forkhead transcription factor FOXO mediates the reduction in cell number associated with reduced insulin signaling. J Biol 2, 20 (2003). 12908874 10.1186/1475-4924-2-20 PMC333403

[b32] Puig O., Marr M. T., Ruhf M. L. & Tjian R. Control of cell number by *Drosophila* FOXO: downstream and feedback regulation of the insulin receptor pathway. Genes Dev 17, 2006–2020 (2003). 12893776 10.1101/gad.1098703 PMC196255

[b33] Kondo S. & Ueda R. Highly improved gene targeting by germline-specific Cas9 expression in *Drosophila*. Genetics 195, 715–721 (2013). 24002648 10.1534/genetics.113.156737 PMC3813859

[b34] Gundelfinger E. D., Hermans-Borgmeyer I., Grenningloh G. & Zopf D. Nucleotide and deduced amino acid sequence of the phosphoenolpyruvate carboxykinase (GTP) from *Drosophila melanogaster*. Nucleic Acids Res 15, 6745 (1987). 3114718 10.1093/nar/15.16.6745 PMC306146

[b35] Yoo S. J. *et al.* Hid, Rpr and Grim negatively regulate DIAP1 levels through distinct mechanisms. Nat Cell Biol 4, 416–424 (2002). 12021767 10.1038/ncb793

[b36] Colombani J. *et al.* Antagonistic actions of ecdysone and insulins determine final size in *Drosophila*. Science 310, 667–670 (2005). 16179433 10.1126/science.1119432

[b37] Pasco M. Y. & Leopold P. High sugar-induced insulin resistance in *Drosophila* relies on the lipocalin Neural Lazarillo. PLoS One 7, e36583 (2012). 22567167 10.1371/journal.pone.0036583 PMC3342234

[b38] Honegger B. *et al.* Imp-L2, a putative homolog of vertebrate IGF-binding protein 7, counteracts insulin signaling in *Drosophila* and is essential for starvation resistance. J Biol 7, 10 (2008). 18412985 10.1186/jbiol72 PMC2323038

[b39] Kim J. & Neufeld T. P. Dietary sugar promotes systemic TOR activation in *Drosophila* through AKH-dependent selective secretion of Dilp3. Nat Commun 6, 6846 (2015). 25882208 10.1038/ncomms7846 PMC4402654

[b40] Kuhnlein R. P. Thematic review series: Lipid droplet synthesis and metabolism: from yeast to man. Lipid droplet-based storage fat metabolism in *Drosophila*. J Lipid Res 53, 1430–1436 (2012). 22566574 10.1194/jlr.R024299 PMC3540836

[b41] Musselman L. P. *et al.* A high-sugar diet produces obesity and insulin resistance in wild-type *Drosophila*. Dis Mod Mechan 4, 842–849 (2011). 10.1242/dmm.007948 PMC3209653 21719444

[b42] Chien C. H., Chen W. W., Wu J. T. & Chang T. C. Label-free imaging of *Drosophila in vivo* by coherent anti-Stokes Raman scattering and two-photon excitation autofluorescence microscopy. J Biomed Opt 16, 016012 (2011). 21280918 10.1117/1.3528642

[b43] Geminard C., Rulifson E. J. & Leopold P. Remote control of insulin secretion by fat cells in *Drosophila*. Cell Metab 10, 199–207 (2009). 19723496 10.1016/j.cmet.2009.08.002

[b44] Ribeiro C. & Dickson B. J. Sex peptide receptor and neuronal TOR/S6K signaling modulate nutrient balancing in *Drosophila*. Curr Biol 20, 1000–1005 (2010). 20471268 10.1016/j.cub.2010.03.061

[b45] Sellami A., Isabel G. & Veenstra J. A. Expression of the mu opioid receptor in *Drosophila* and its effects on trehalose and glycogen when expressed by the AKH neuroendocrine cells. Peptides 31, 1383–1389 (2010). 20420874 10.1016/j.peptides.2010.04.015

[b46] Clark L., Lange A. B., Zhang J. R. & Tobe S. S. The roles of Dippu-allatostatin in the modulation of hormone release in *Locusta migratoria*. J Insect Physiol 54, 949–958 (2008). 18479700 10.1016/j.jinsphys.2008.03.007

[b47] Van der Horst D. J. Insect adipokinetic hormones: release and integration of flight energy metabolism. Comp Biochem Physiol B - Biochem Mol Biol 136, 217–226 (2003). 14529748 10.1016/s1096-4959(03)00151-9

[b48] Yoon J. G. & Stay B. Immunocytochemical localization of *Diploptera punctata* allatostatin-like peptide in *Drosophila melanogaster*. J Comp Neurol 363, 475–488 (1995). 8847412 10.1002/cne.903630310

[b49] Dimitriadis G., Mitrou P., Lambadiari V., Maratou E. & Raptis S. A. Insulin effects in muscle and adipose tissue. Diabetes Res Clin Pract 93 Suppl 1, S52–59 (2011). 21864752 10.1016/S0168-8227(11)70014-6

[b50] Felig P., Wahren J., Sherwin R. & Hendler R. Insulin, glucagon, and somatostatin in normal physiology and diabetes mellitus. Diabetes 25, 1091–1099 (1976). 992227 10.2337/diab.25.12.1091

[b51] Unger R. H., Ohneda A., Aguilar-Parada E. & Eisentraut A. M. The role of aminogenic glucagon secretion in blood glucose homeostasis. J Clin Invest 48, 810–822 (1969). 5780193 10.1172/JCI106039 PMC322289

[b52] Gulia-Nuss M., Robertson A. E., Brown M. R. & Strand M. R. Insulin-like peptides and the target of rapamycin pathway coordinately regulate blood digestion and egg maturation in the mosquito *Aedes aegypti*. PLoS One 6, e20401 (2011). 21647424 10.1371/journal.pone.0020401 PMC3103545

[b53] Brown M. R. *et al.* An insulin-like peptide regulates egg maturation and metabolism in the mosquito *Aedes aegypti*. Proc Natl Acad Sci USA 105, 5716–5721 (2008). 18391205 10.1073/pnas.0800478105 PMC2311378

[b54] Toshima N. & Tanimura T. Taste preference for amino acids is dependent on internal nutritional state in *Drosophila melanogaster*. J Exp Biol 215, 2827–2832 (2012). 22837455 10.1242/jeb.069146

[b55] Bendena W. G. *et al.* A *Caenorhabditis elegans* allatostatin/galanin-like receptor NPR-9 inhibits local search behavior in response to feeding cues. Proc Natl Acad Sci USA 105, 1339–1342 (2008). 18216257 10.1073/pnas.0709492105 PMC2234139

[b56] Diao F. & White B. H. A novel approach for directing transgene expression in *Drosophila*: T2A-Gal4 in-frame fusion. Genetics 190, 1139–1144 (2012). 22209908 10.1534/genetics.111.136291 PMC3296248

[b57] Rajan A. & Perrimon N. *Drosophila* cytokine unpaired 2 regulates physiological homeostasis by remotely controlling insulin secretion. Cell 151, 123–137 (2012). 23021220 10.1016/j.cell.2012.08.019 PMC3475207

[b58] Danielsen E. T. *et al.* Transcriptional control of steroid biosynthesis genes in the *Drosophila* prothoracic gland by Ventral veins lacking and Knirps. PLoS Genet 10, e1004343 (2014). 24945799 10.1371/journal.pgen.1004343 PMC4063667

[b59] Deshpande S. A. *et al.* Quantifying *Drosophila* food intake: comparative analysis of current methodology. Nat Methods 11, 535–540 (2014). 24681694 10.1038/nmeth.2899 PMC4008671

