# Supplementary material for: The Neuropeptide Allatostatin A Regulates Metabolism and Feeding Decisions in Drosophila
Source: Sci Rep. 2015 Jun 30;5:11680. doi: 10.1038/srep11680 (PMC4485031; doi:10.1038/srep11680)
Supplement: Supplementary Information [file srep11680-s1.doc]

## Supplementary Information

# The Neuropeptide Allatostatin A Regulates Metabolism and Feeding Decisions in *Drosophila*

Julie L. Hentze, Mikael A. Carlsson, Shu Kondo, Dick R. Nässel, Kim F. Rewitz


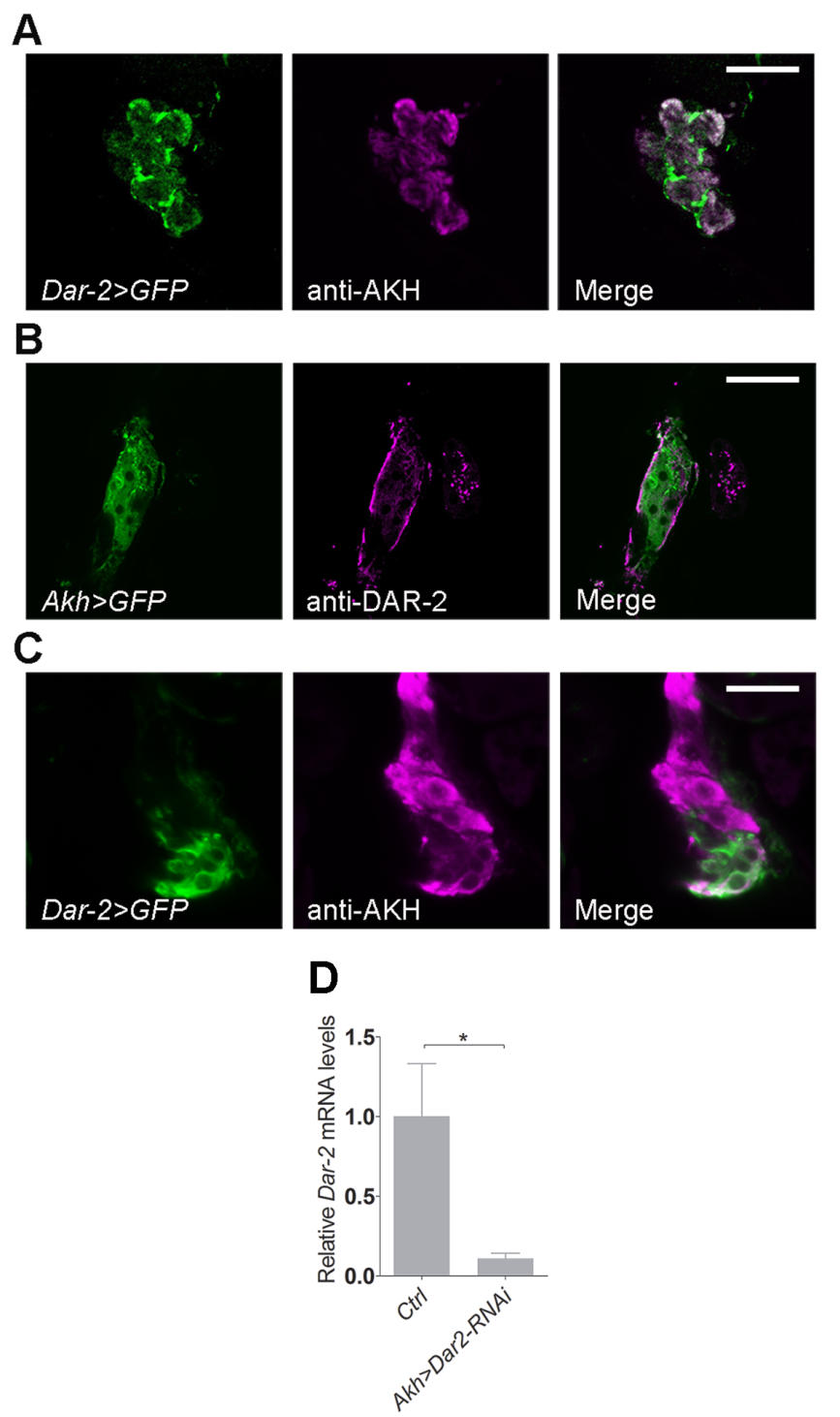


**Figure S1. Expression of *Dar-2* in the APCs.** (A) 3^rd^ instar larvae. (B-C) adult. (A + C) Colocalization of GFP expression, driven by *Dar-2>* (green) and anti-AKH immunostaining (magenta). (B) Co localization of GFP expression driven by *Akh>* (green) and anti-DAR-2 immunostaining (magenta). (D) Analysis of the expression of *Dar-2* in the CC from *Akh>GFP/+* control (Ctrl) animals and *Akh>GFP*,*Dar-2-RNAi* flies where *Dar-2* had been silenced using the CC-specific *Akh>* driver, shows that *Dar-2* is efficiently knocked down, demonstrating *Dar-2* expression in the adult CC. Scale bars, 15 µM in A,C and 20 µM in B. Error bars indicate standard errors (n=3). *: *P* < 0.05 (Student’s t-test).


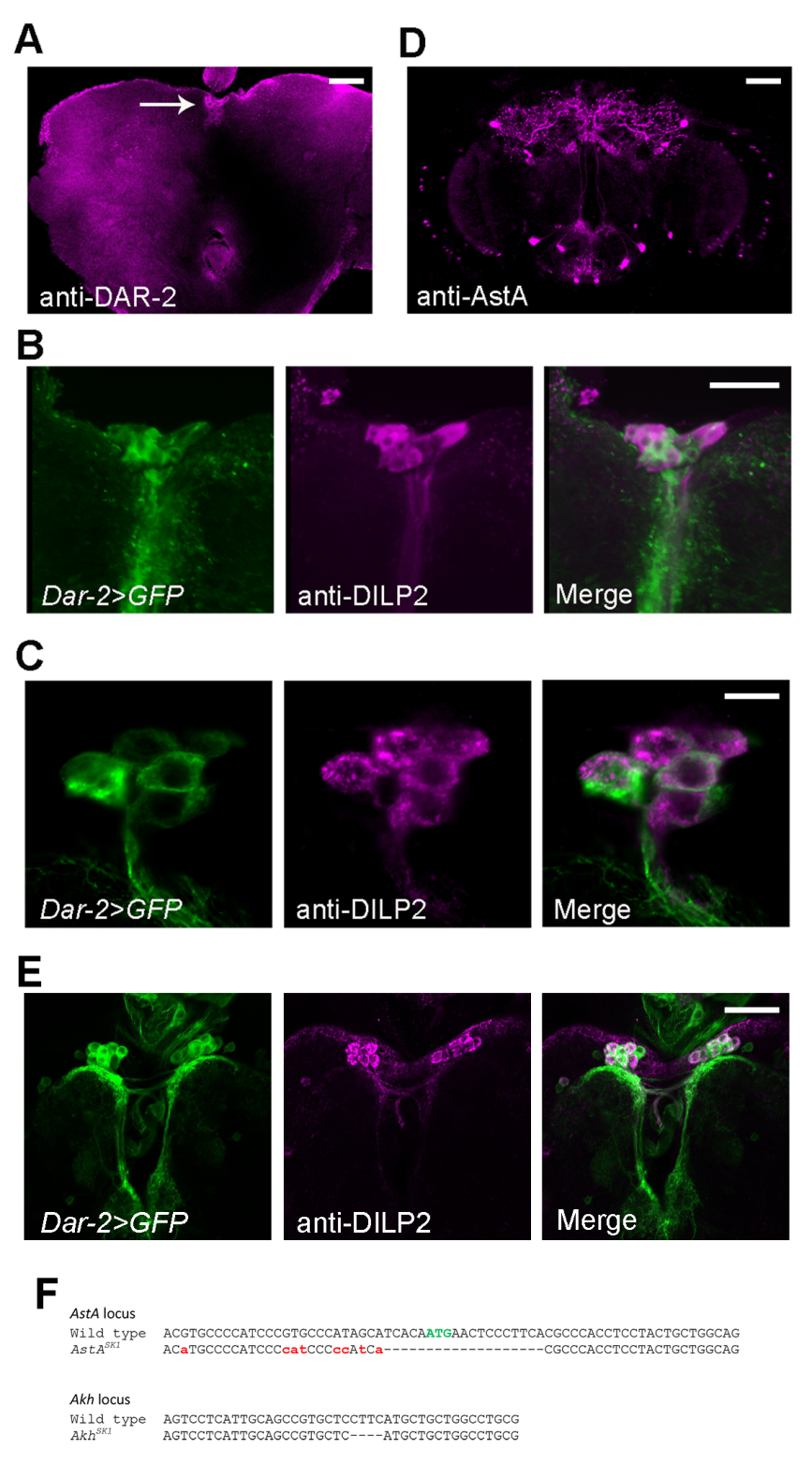


Figure S2. The AstA producing neurons and *Dar-2* expression in the IPCs of larval and adult brains. (A) Anti-DAR-2 immunostaining (magenta) in the adult brain. White arrow indicates the IPCs. (B-C) Colocalization of GFP expression driven by *Dar-2>* (green) and anti-DILP2 immunostaining (magenta) in adult (B) and 3^rd^ instar larvae (C). (D) Anti-AstA immunostaining (magenta) in the adult brain. (E) The CRISPR/Cas9 induced *Gal4* reporter knock-in the endogenous *Dar-2* gene drives expression of *GFP* (green) in DILP2 positive cells of the larval brain (magenta), demonstrating *Dar-2* expression in the IPCs. (F) Sequences of Cas9-induced mutations in the *AstA* and *Akh* genes. Deleted nucleotides are shown as dashes, while mutated nucleotides are shown in red and green indicates the ATG start codon. Scale bars, 40 µM in A,B,E, 10 µM in C and 50 µM in D.


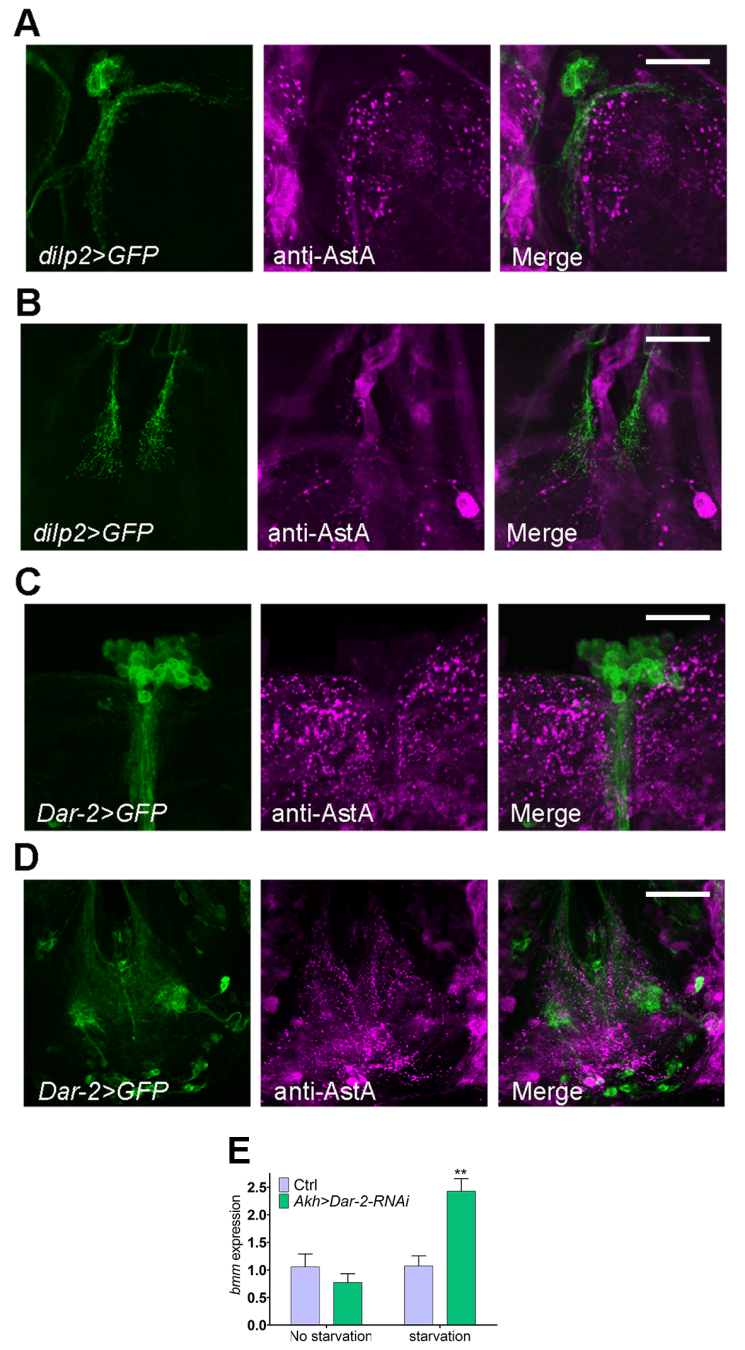


Figure S3. AstA neurons arborize in the proximity of the IPCs in larval and adult brains. (A-D) Localization of GFP expression driven by *dilp2>* in 3^rd^ instar larvae (green) (A-B) or the driver *Dar-2>* in adult (green). (C-D) and anti-AstA immunostaining (magenta) in the protocerebrum (A,C) and SOG (B,D). The neural processes shown in B are derived from the same cells shown in A and those shown in D are derived from the same cells shown in C. (E) Expression of *brummer* (*bmm*) in adult males kept under standard food condition (no starvation) or after 6 hours starvation. Scale bars, 40 µM. **: *P* < 0.01 (Student’s t-test).

## Table S1: qPCR primers

| *F1:4EBP* | CCAGGAAGGTTGTCATCTCG |
| --- | --- |
| *R1:4EBP* | CCAGGAGTGGTGGAGTAGAGG |
| *F1:dilp3* | CAACGCAATGACCAAGAGAAC |
| *R1:dilp3* | GCATCTGAACCGAACTATCACTC |
| *F1:dilp2* | CAGGAGTTCGAGGAGGAGGA |
| *R1:dilp2* | AAGATAGCTCCCAGGAAAGAGG |
| *F1:tobi* | CCACCAAGCGAGACATTTACC |
| *R1:tobi* | GAGCGGCGTAGTCCATCAC |
| *F1:Akh* | AGACCTCCAACGAAATGCTG |
| *R1:Akh* | GTGCTTGCAGTCCAGAAAGAG |
| *F1AKHR* | CACACCTCGCTGTCCAATC |
| *R1AKHR* | CATCACCTGGCCTCTTCCA |
| *F1:PEPCK* | TCAATGGCGAATCCTGCTAC |
| *R1:PEPCK* | TCCTTCACGTCCACCTTATCC |
| *F1:Dar-2* | GAGACGAACACCCTCACCAA |
| *R1:Dar-2* | AGCAGCGGATTGATACACGA |
| *F1:AstA* | GAGGTCTCGTCCCTACTCCTTC |
| *R1:AstA* | GATCTCGTTGTCCTGGTCGT |
| *F1:bmm*  *R1:bmm* | GGTCCCTTCAGTCCCTCCTT  GCTTGTGAGCATCGTCTGGT |
| *F1:rpL23* | GACAACACCGGAGCCAAGAACC |
| *R1:rpL23* | GTTTGCGCTGCCGAATAACCAC |
